# Nurse practitioners’ implementation assessment in Swiss nursing homes: A mixed study protocol (the EVALIPS_VD Project)

**DOI:** 10.1016/j.ijnsa.2026.100526

**Published:** 2026-03-31

**Authors:** Ricardo Salgado, Nishalini Gunalingam, Renée Dion, Hélène Chenevey-Antoine, Claudia Ortoleva Bucher

**Affiliations:** a La Source School of Nursing, HES-SO University of Applied Sciences and Arts Western Switzerland, Lausanne, Switzerland; b Fondation Belle Saison, Mont-sur-Rolle, Switzerland; c Fondation Pré Pariset, Pully, Switzerland; d HévivA - Association vaudoise des institutions médico-psycho-sociales, Renens, Switzerland; e School of Health Sciences Fribourg, HES-SO University of Applied Sciences and Arts Western Switzerland, Fribourg, Switzerland

**Keywords:** Nurse practitioners, Nursing homes, Implementation science, Evaluation

## Abstract

**Background:**

Switzerland faces challenges in long-term care due to its high life expectancy and the resulting increased prevalence of non-communicable diseases and functional dependence among older adults. Nursing homes are addressing increasingly complex acute care needs amid healthcare professional shortages. The nurse practitioner role has emerged globally as a key strategy to enhance the accessibility, quality, and coordination of care in these settings. While the role was recently legislated in the canton of Vaud, there's a scarcity of solid scientific data on nurse practitioner implementation and impact in Switzerland. The aim of this study is to assess the implementation of the nurse practitioner role in nursing homes in order to develop a toolkit that supports the sustainability of this new role’s deployment in Switzerland.

**Methods and analysis:**

This two-phase study will evaluate the implementation of the nurse practitioner role in two distinct nursing homes in the canton of Vaud, using the PEPPA Plus model (based on Donabedian's framework) as a guide. Phase 1 employs an embedded mixed-method case study design across three work packages (WPs). WP1 uses Rapid Ethnography (observations, interviews, document analysis) to assess structural elements influencing implementation before and one year after nurse practitioner deployment. WP2 uses a prospective descriptive quantitative design where NPs log their activities per act daily or five consecutive days each month over the course of one year. WP3 uses a longitudinal multi-method approach, combining a pre-post quasi-experimental quantitative component to measure resident, staff, and institutional outcomes (e.g., hospitalizations, pressure sores) at 3, 6, and 12 months, with qualitative interviews. Phase 2 (WP4) will use Thoele's three-step methodology—data preparation, focus group evaluation, and toolkit design—to develop a best practices toolkit to support the nurse practitioner role's sustainability based on Phase 1 results. For Phase 1 analysis, data from each nursing home will be analyzed separately (intra-case analysis) and then compared (inter-case analysis). Qualitative data (interviews) will be analyzed using thematic content analysis and triangulated with quantitative data for a comprehensive understanding. Quantitative outcomes will be analyzed using descriptive statistics and generalized linear models (e.g., Poisson regression) for pre-post comparisons over two six-month periods.

**Ethics and dissemination:**

All procedures will comply with the ethical principles outlined in the Declaration of Helsinki. Given the nature of the study, ethical approval from the Ethics Commission of the Canton of Vaud was not required, according to the latter. Data and material will be available on request from the authors.

**Trial registration number:**

10.17605/OSF.IO/7FX8M

**What is already known**

• Nurse practitioner roles have been introduced in nursing homes in several countries to respond to increasing care complexity and workforce shortages.
• Existing studies suggest that nurse practitioners may improve care quality, care coordination, and healthcare use in long-term care settings.
• However, evidence on the implementation of nurse practitioner roles in nursing homes remains limited, particularly in Switzerland.

Alt-text: Unlabelled box dummy alt text

**What this paper adds**

• This protocol describes a mixed-method study designed to examine the implementation of the nurse practitioner role in two Swiss nursing homes.
• The study will document structural conditions, nurse practitioner activities, and outcomes related to residents, healthcare professionals, and institutions over time.
• The findings will inform the development of a toolkit to support the implementation and sustainability of nurse practitioner roles in nursing homes.

Alt-text: Unlabelled box dummy alt text

## 1 Introduction

Switzerland is one of the countries in the world with the highest life expectancy (Office fédérale de la statistique, 2024). This demographic phenomenon is accompanied by a high prevalence of non-communicable diseases, associated with a decrease in physiological reserves, an increase in functional dependence and a higher incidence of neurocognitive disorders (WHO, 2024). This reality increases the complexity and intensity of the care required by these individuals, making the need for continuous, tailored and coordinated care even more crucial. This care is often provided in long-term care facilities such as nursing homes, where resources and specialised skills are essential to meet the ageing challenges (European Commission, 2024). As a result, nursing homes are facing increasingly complex and acute health situations, exacerbated by a shortage of healthcare professionals, which represents a major challenge (Favez and Zúñiga, 2021). Furthermore, increasing pressure on healthcare systems is leading to more frequent and rapid hospital transfers of older people in poorer health to nursing homes. This situation threatens the quality, safety and continuity of care (Katz and Karuza, 2005).

In response to these challenges, various innovative care models have been developed to improve the accessibility and quality of care provided within the healthcare system, including in nursing homes, notably through the integration of nurse practitioners (Schober *et al.*, 2020; Gentizon *et al.*, 2024). According to Hamric, their core competencies include direct clinical practice, guidance and coaching, evidence-based practice, consultation, clinical leadership, interprofessional collaboration, and ethical decision-making support (Schober *et al.*, 2020; Gentizon *et al.*, 2024). Nurse practitioners focus on providing direct patient care, with a particular emphasis on health promotion and self-management of chronic conditions. (Schober *et al.*, 2020; Kilpatrick *et al.*, 2023, 2024; Brownwood and Lafortune, 2024). They are authorised to carry out in-depth clinical assessments, prescribe laboratory tests or other diagnostic tests, and even prescribe medicines and treatments, depending on the regulatory context (Schober *et al.*, 2020; Gentizon *et al.*, 2024). They usually work independently or in collaboration with other healthcare professionals (such as physicians), for example to discuss complex clinical situations (Schober *et al.*, 2020; Gentizon *et al.*, 2024). With their in-depth knowledge and skills, nurse practitioners improve: efficiency in the organisation of healthcare system resources (reducing emergency room visits, hospital transfers and costs), chronic disease management, mortality rates and satisfaction among residents, their families and other healthcare workers (Craswell *et al.*, 2020; National Academies of Sciences, Engineering, and Medicine; National Academy of Medicine; Committee on the Future of Nursing 2020–2030, 2021; *The National Imperative to Improve Nursing Home Quality: Honoring Our Commitment to Residents, Families, and Staff*, 2022; Alexander *et al.*, 2023; Gentizon *et al.*, 2024).

In 2020, the role of nurse practitioners was legislated in the canton of Vaud, which remains to date the only canton in Switzerland to have established specific regulations governing their practice (Gyuriga Perez and Peguet, 2024). The authorities in this canton are currently supporting the first nurses undergoing master's degree training as nurse practitioners, two of whom had begun working in two nursing homes in October 2025. In this emerging context, which is also marked by a lack of solid scientific data in Switzerland and internationally, it seems essential to evaluate the process of implementing nurse practitioners in nursing homes and the added value of their role. This study will provide data on implementation process, the role and impact of nurse practitioners in two nursing homes in Switzerland, contribute to efforts to roll out the programme at cantonal and national level and support the long-term sustainability of the role through the development of a practical toolkit.

The PEPPA Plus model (Participatory, Evidence-based, Patient-focused Process for Advanced Practice Nursing) that will guide this study, was developed in Switzerland in 2016 and is based on the PEPPA model originally developed by Bryant-Lukosius and DiCenso in 2004 (Bryant-Lukosius and DiCenso, 2004). It provides a detailed and operational framework to support the assessment of the various factors influencing the deployment of the role of advanced practice nurses, such as clinical nurses specialists and nurse practitioners, in various clinical contexts (Rogers *et al.*, 2024). Its goal is to contribute to improving the health outcomes of patients and their families by ensuring high-quality, patient-centred and economically sustainable care.

It is based on Donabedian's evaluation framework (Donabedian, 1966) which lists the elements to be assessed during the introduction, implementation and sustainability phases, namely: (1) existing structures (political, organisational, cultural, economic, human resources and logistical elements); (2) processes put in place (use of the scope of practice and timing and dosage of Advanced Practice Nurse activity); and (3) outcomes (at the level of patients and their families, quality of care, Advanced Practice Nurse and various stakeholders, organisation and use of health system resources).

## 2 Method and analysis

The reporting of this study follows the StaRI guidelines for implementation studies (Pinnock *et al.*, 2017).

To achieve the project's goal, two phases have been established.

The overall objective of Phase 1 is to evaluate the implementation of a nurse practitioner in nursing homes and the effects of this new role on indicators related to residents, other healthcare workers and the institution.

Phase 1 consists of three work packages (WPs) with the following respective objectives: (1) WP1: Assess the structural elements that potentially influence the implementation of the nurse practitioner role; (2) WP2: Explore and describe the activities carried out by nurse practitioners within the nursing home; (3) WP3: Understand the influence of nurse practitioner implementation elements on the evolution of residents, health care workers and the institution outcomes, that are sensitive to nurse practitioner role.

Phase 2 (consisting of WP4) aims to develop a toolkit listing best practices to support the sustainability of the nurse practitioner role, based on the results obtained in Phase 1.

The two participating nursing homes have their own organisational structures and will be considered as separate cases.

To meet the objectives of Phase 1, we will use an embedded mixed method case study design (Creswell and Plano Clark, 2018). To meet the objectives of Phase 2, we will use Thoele's three-step methodology (Thoele *et al.*, 2020). Table 1 summarises the objectives of the two phases and the four WPs, as well as the methodologies that will be used.

**Table 1:** Specific objectives of the study, its WPs and associated estimates.

Phase aim	Design/methods	WP	Objectives	WP design/WP methods
Phase 1: Evaluate the implementation of a nurse practitioner in nursing homes and the effects of this new role on residents, healthcare workers and institution outcomes	Embedded mixed method case study (Creswell and Plano Clark, 2018)	1	Assess the structural elements that potentially influence the implementation of the nurse practitioner role	Rapid ethnograpy (Vindrola-Padros and Vindrola-Padros, 2018)
		2	Describe the activities per act carried out by nurse practitioners within the nursing home	Prospective, descriptive, quantitative design
		3	Understand the influence of nurse practitioner implementation elements on the evolution of residents, health care workers and the institution outcomes, that are sensitive to nurse practitioner role	Longitudinal multi-method: quasi-experimental pre-post and qualitative interviews
Phase 2: Develop a toolkit listing best practices to support the sustainability of the nurse practitioner role, based on the results obtained in Phase 1	Thoele's three-step methodology (Thoele *et al.*, 2020)	4	-	-

### 2.1 Phase 1 methods

This section presents the first three WPs methodologies, followed by the analysis plan for phase 1, namely the embedded mixed method case study. (Creswell and Plano Clark, 2018)

*WP1 objective:* Assess the structural elements that potentially influence the implementation of the nurse practitioner role before the nurse practitioner implementation and one year after implementation*.*

*WP1 design and method:* To assess the structural elements that could potentially influence the implementation of the nurse practitioner role, a contextual analysis will be carried out using the Rapid Ethnography approach. (Vindrola-Padros and Vindrola-Padros, 2018). Rapid Ethnography is a research approach that adapts traditional ethnographic methods to produce results in shorter periods, while maintaining depth of analysis on social processes and context. It is characterised by intensive data collection over a brief period, the use of multiple data collection methods (observations, logbooks, institutional documents, and interviews), iterative analysis parallel to data collection, and the triangulation of several types of data.

*WP1 data collection*: Each nursing home will be visited for three days before the nurse practitioner role implementation and one year after implementation. Data collection covers all factors that could potentially influence the implementation of the nurse practitioner role: (1) organisation and culture of the establishment (e.g. number of residents, nurse practitioner specifications, institution values and priorities); (2) perception of the nurse practitioner role (planned organisation, relevance, clarity, support and acceptance of the role) by the various stakeholders (nurse practitioner, medical and nursing professionals, managers, residents and families); (3) human resources (e.g. full-time equivalent, team composition); and (4) technical and practical resources (e.g. consultation room, nurse practitioner support – mentoring, training). Observers visiting nursing homes will have observation notebooks in which they will record all their observations. For the semi-structured interviews (on all the topics described above), at least one manager, one clinical nurse specialist, one nurse, one community health care assistant, one nursing assistant, one physician and one resident and their family member, as predetermined by the nursing home management, will be included per nursing home. A total of fourteen interviews will be conducted (seven per nursing home) in a confidential location. Each interview will be digitally recorded and transcribed using Whisper software, then uploaded to MAXQDA for analysis. Relevant institutional documents will also be collected for analysis. A logbook (personal notes and field notes) will supplement the qualitative data. Conducting the study one year after implementation will provide insight into how the various elements assessed prior to the role's implementation have evolved.

#### 2.1.1 WP1 participants

Inclusion criteria for nurse practitioner, healthcare workers and managers: All individuals present during visits and predetermined by the nursing home management are invited to participate. The inclusion criteria are as follows: (1) consent to participate in the study, (2) have a good understanding of French. Exclusion criteria: Students, temporary staff, and staff hired less than three months ago.

Inclusion criteria for residents and families: All residents and families present during visits and predetermined by the nursing home management are invited to participate. The inclusion criteria are as follows: (1) consent to participate in the study, (2) have the capacity for discernment, (3) have a good understanding of French. Exclusion criterion: having a diagnosed severe cognitive impairment.

*WP1 Data analysis:* Qualitative data from the interviews will be analysed using thematic content analysis according to Braun and Clarke (2022) and triangulation with data collected through observation and institutional documents in order to identify information, relationships and meanings in the data relating to elements that could potentially influence the implementation of the nurse practitioner role (Larivière and Corbière, 2014). These analyses will be conducted using MAXQDA 2022 text analysis software. Verbatim excerpts will illustrate trends and allow the phenomenon of implementation to be recontextualised.

*WP2 objective:* Describe the activities conducted by nurse practitioners within the nursing home.

*WP2 design and method:* Prospective descriptive quantitative design. To list the activities conducted per act by the nurse practitioner, the latter will systematically compile its activities after each activity.

*WP2 data collection:* Over five consecutive days each month across a 12-month period, nurse practitioners will complete a computerized form (Microsoft Forms) immediately following each act performed during their workday. The form will record the date, the mean duration of the act, and the type(s) of activity performed during the act, selected from a standardized drop-down menu. This menu with 37 different activities was developed specifically for the purposes of this study and is based on the validated list of nurse practitioner activities developed by Kilpatrick (2011) and previously used in long-term care settings (Landry *et al.*, 2020). This classification captures the full scope of nurse practitioner practice, encompassing direct and indirect clinical care as well as non-clinical activities related to education, research, and administrative responsibilities. In addition, the form will document the source of the nurse practitioner solicitation, the presence of beneficiaries (residents and/or family members), and the involvement of other healthcare or social care professionals in interprofessional collaboration (e.g., physicians, physiotherapists).

*WP2 participants*: nurse practitioners active in both participating nursing homes.

*WP2 data analysis:* Descriptive analyses will be computed on a daily, weekly, and monthly basis: average duration, total duration (by act, activity, by persons present); average number and total number of activities carried out, and skills mobilised.

*WP3 objective:* Understand the influence of nurse practitioners implementation on the evolution of residents, health care workers, and the institution outcomes, which are sensitive to nurse practitioner role.

*WP3 design and method:* We will use a convergent multi-method approach combining a quantitative component, consisting of a pre–post comparative study of outcomes sensitive to the nurse practitioner role, with a qualitative component based on semi-structured interviews exploring stakeholders’ perceptions and experiences of the implementation of the role.

*WP3 data collection:* For the quantitative component, outcomes will be measured six months prior to the implementation of the nurse practitioner role and at 3 (T1), 6 (T2), and 12 (T3) months after implementation in each nursing home. The qualitative component will be conducted at 3 (T1), 6 (T2), and 12 (T3) months after the implementation of the nurse practitioner role in each nursing home.

*Quantitative outcomes:*(a) Quality of care, assessed through the incidence of falls; prevalence and incidence of pressure ulcers; prevalence and incidence of physical restraints; and nutritional status; and (b) use of the healthcare system, measured by the number of hospital admissions; the rate of unplanned 30-day readmissions related to previously identified conditions; and the number of unscheduled medical solicitations outside routine medical visits.

*Qualitative outcomes* explored stakeholders’ experiences and perceptions of the implementation of the nurse practitioner role and were structured across two participant groups: (a) Residents and families: quality and satisfaction with overall care; participation in decision-making; perceptions of a patient-centered approach and of safety; and perceptions of the clarity, relevance, and acceptance of the Nurse Practitioner role; (b) Nurse practitioners, healthcare professionals, and institutional stakeholders: satisfaction with the nurse practitioner role; perceived relevance and clarity of the role and its organizational integration; acceptance of the role; perceived barriers and facilitators to implementation; and experiences and perceptions of interprofessional collaboration.

#### 2.1.2 WP3 Measuring instruments

The quantitative data will be extracted from data routinely collected by nursing homes as part of quality indicators and available in computerised records.

*WP3 participants:* Identical to those of WP1. Qualitative data will be collected during semi-structured interviews. At least one manager, one clinical nurse specialist, one nurse, one healthcare assistant, one nursing assistant, one physician, one resident, and one family member predetermined by the nursing home management will be included per nursing home. A total of fourteen interviews will be conducted (seven per nursing home) in a confidential location. The estimated duration of each interview is 30 minutes. Each interview will be digitally recorded and transcribed using Whisper software, then uploaded to MAXQDA 2022 for analysis.

#### 2.1.3 WP3 data analysis plan

Quantitative data: Descriptive statistics (frequencies, means, standard deviations, medians and confidence intervals) will be computed. The characteristics of the nursing homes and the demographic characteristics of the participants will be summarised using frequencies and proportions for nominal variables and means and standard deviations for continuous variables. For pre-post comparisons of quantitative data on quality of care and health system utilisation, periods of 6 months before the implementation of the Nurse Practitioner will be considered. Analyses will be conducted for each unit separately, using generalized linear models (mainly Poisson, quasi-Poisson or negative binomial regressions).

Qualitative data: Qualitative data from the interviews will be analysed using thematic content analysis according to Braun and Clarke (2022). These analyses will be carried out using MAXQDA 2022® text analysis software. Verbatim excerpts will illustrate trends and provide context for the phenomenon of implantation. Guba and Lincoln's criteria for rigour (Larivière and Corbière, 2014) will be adopted to ensure the scientific quality of the qualitative design

#### 2.1.4 Phase 1 analysis plan: embedded mixed method case study

**Fig. 1:**
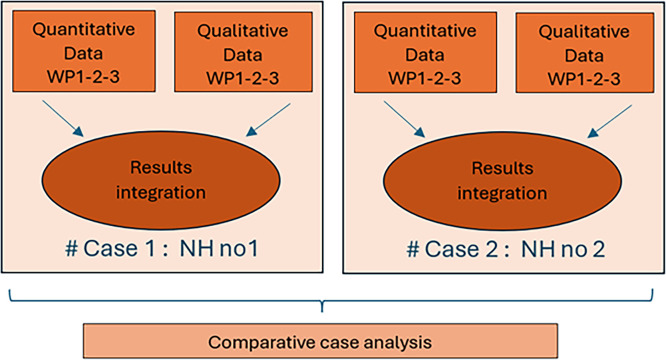
Embedded mixed method case study design.

As a reminder, each nursing home will be considered as a unique case. The data will be analyzed separately for each case (intra-case analysis), then the cases will be compared with each other (inter-case analysis) (Fig. 1).

Intra-case analysis: For each nursing homes, the data will be analysed in two steps: (1) concurrent analysis of quantitative data on the one hand and qualitative data on the other, and (2) integration of quantitative and qualitative data. The intra-case analysis will provide a comprehensive description of the Nurse Practitioner implementation trajectory for each case (each nursing home). Quantitative and qualitative analysis: the results of the quantitative and qualitative analyses of WPs 1, 2 and 3 will form the basis of the mixed analysis. Qualitative data will be combined with quantitative data through triangulation. The results derived from both types of data will be compared and discussed using a side-by-side approach to determine whether they converge or diverge and how they complement each other to meet the study objectives (Creswell and Plano Clark, 2018). Qualitative data complement and clarify quantitative data.

Inter-case analysis (Fig. 1): The results of the analyses for each case will be compared in order to highlight similarities and differences, attempt to explain them, and explore possible causal links (Creswell and Plano Clark, 2018). The results as a whole will answer the research questions and provide an overview of the outcomes of the implementation strategies.

### 2.2 Phase 2 methods

*WP4 objective:* develop a toolkit listing best practices to support the sustainability of the nurse practitioner role, based on the results obtained in Phase 1. This toolkit will be used to guide the project's partner nursing homes. It may also be used by other nursing homes that implement the nurse practitioner role. *WP design and method:* The methodology proposed by Thoele et al. will be used (Thoele *et al.*, 2020), based on the results of Baumann's exploratory study (Baumann *et al.*, 2022). This methodology comprises three steps: (1) preparation of the toolkit data; (2) evaluation and discussion; and (3) design of the toolkit.

Step 1 consists in the preparation of toolkit data: Researchers will merge the data collected throughout the implementation process during Phase 1 (and its three WPs). Researchers will prepare a summary that will be sent to stakeholders at each participating site for individual reflection. Step 2 consists in evaluation and discussion: Researchers and coordinators from the two nursing homes participating in the study are invited to a focus group at the end of data collection for phase 1 (and its three WPs). This focus group includes the various stakeholders from each participating unit: senior management, the unit manager, the clinical nurse specialist and the nurse practitioners, and other healthcare workers. The discussion will be guided by a research teams members based on the summaries previously sent to participants. As a group, stakeholders will reflect on the tools and implementation strategies that have proven effective and relevant. They will also consider how to improve the toolkit with regard to strategies that have proven less effective. Step 3 consists in toolkit design: Researchers are compiling the discussions from stage 2 to create the final toolkit in the form of a collection of documents designed for nursing homes and aimed at informing and facilitating the sustainability of an nurse practitioner in nursing homes.

### 2.3 Project schedule

**Fig. 2:**
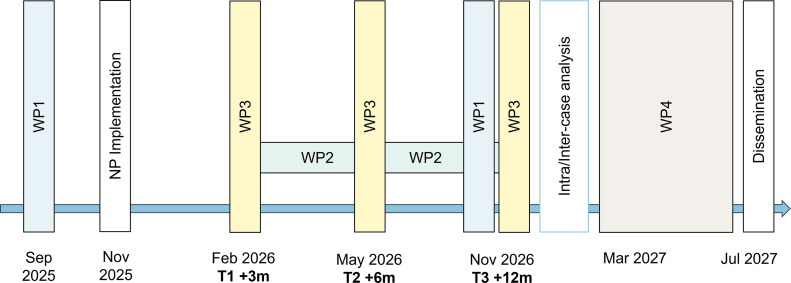
Main steps of the project and timeline for the various WPs.

The main steps of the project and the forecast for their completion are presented in Fig. 2.

## Ethics and dissemination

All procedures will comply with the ethical principles outlined in the Declaration of Helsinki. Given the nature of the study, ethical approval from the Ethics Commission of the Canton of Vaud was not required, according to the latter. Data and material will be available on request from the authors.

We will share our findings through multiple channels, tailored to different audiences. We will present at public conferences within institutions and publish scientific manuscripts in international and French journals. Additionally, we will present at relevant local and international conferences. Authorship will adhere to the International Committee of Medical Journal Editors' guidelines

## Patient and public involvement

Patients and/or the public were not involved in the design, conduct, reporting or dissemination plans of this research.

## Declaration of generative AI and AI-assisted technologies in the manuscript preparation process

During the preparation of this work, the authors used ChatGPT to improve the clarity and style of the text of the manuscript. After using this tool, the authors reviewed and edited the content as needed and take full responsibility for the content of the published article.

## Patient consent for publication

Not required.

## Funding

This study is funded by the Health Domain grant from the University of Applied Sciences and Arts Western Switzerland (11M25).

## CRediT authorship contribution statement

**Ricardo Salgado:** Conceptualization, Funding acquisition, Methodology, Validation, Writing – original draft, Writing – review & editing. **Nishalini Gunalingam:** Writing – review & editing. **Renée Dion:** Writing – review & editing. **Hélène Chenevey-Antoine:** Writing – review & editing. **Claudia Ortoleva Bucher:** Conceptualization, Funding acquisition, Methodology, Validation, Writing – original draft, Writing – review & editing.

## Declaration of competing interest

The authors declare the following financial interests/personal relationships which may be considered as potential competing interests: Ricardo Salgado and Claudia Ortoleva Bucher reports financial support was provided by Health Domain grant from the University of Applied Sciences and Arts Western Switzerland. The other authorsdeclare that they have no known competing financial interests or personal relationships that could have appeared to influence the work reported in this paper.

## Appendix Supplementary materials

Image, application 1

Image, application 2
